# Stroke mimics in patients with clinical signs of stroke

**DOI:** 10.22088/cjim.8.3.213

**Published:** 2017

**Authors:** Mozafar Hosseininezhad, Reza Sohrabnejad

**Affiliations:** 1 Department of Neurology, Poursina Hospital, Guilan University of Medical Sciences, Rasht, Iran.; 2 Mobility Impairment Research, Health Research Institute, Babol, University of Medical Sciences, Babol, Iran.; 3 Department of Neurology, Ayatollah Rouhani Hospital, Babol University of Medical Sciences, Babol, Iran.

**Keywords:** Frequency, Brain stroke, Stroke mimic

## Abstract

**Background:**

Stroke mimic is a major diagnostic challenge and may be difficult to distinguish from real strokes. The aim of this study was to evaluate the relative frequency of stroke mimics in patients with clinical signs of stroke.

**Methods:**

In this cross sectional-study, the medical records of 1985 patients with stroke admitted to Poursina Hospital were enrolled using the census technique. Data collection tool was a checklist which include age, sex, imaging results (MRI and CT scan and primary and final diagnoses.

**Results:**

Of the 1985 patients, 295 (14.9%) were identified with brain stroke mimics. The mean age in the group of patients with mimics and real stroke were 66.5±16.4 years and 72.4±9.6 years, respectively. The highest number of stroke belonged to 61-80 years in stroke groups (68.8%) and mimics (58.3%), respectively. There was a significant correlation between age and early diagnosis of stroke or stroke mimic (P<0.0001). The highest frequency of stroke mimics was related to brain tumors (10.5%), hypoglycemia (9.2%) and toxic poisoning (8.5%).

**Conclusion:**

Due to the high number of stroke mimics, further attention is necessary to aid in differential diagnosis and clinical procedures in patients with stroke signs.

**B**rain stroke is the second cause of death and ranks as the sixth among the most common diseases worldwide (1). Various studies have been carried out in Iran which reported different rates of incidence of stroke (2). The term stroke means sudden occurrence of syndromes and focal neurological signs for more than 24 hours due to prior cerebrovascular problems (3). This is an acute circumstance which is best diagnosed in the shortest duration of time based on patient's report, clinical examination and testing tools as well as imaging brain techniques, (CT or MRI) to considerably reduce mortality and complications through prompt interventions (4). Certain disorders can mimic clinical signs of real stroke. Thus, primary mistaken diagnosis and delayed or inappropriate treatment may cause real stroke complications (4). In various studies, the most common stroke mimics include brain tumors (gliomas, meningiomas, and adenomas are the most common ones) (4), toxic or metabolic disorders (such as hypoglycemia, hypercalcemia, hyponatremia, uremia, hepatic encephalopathy, hyperthyroidism, thyroid storm (4-6), infectious disorders (e.g. meningoencephalitis) (6), psychological disorders and migraines, seizures (5, 6), and demyelization disorders (5). Although such occasions physiologically differ from brain strokes, similiraty of symptoms make the diagnosis difficult (5, 6). 

Lack of proper and timely diagnosis of stroke or stroke mimic can lead to irreversible complications which made us work on this important issue with somewhat different reports from those of other countries.

## Methods

In this cross-sectional study with census method, all patients presenting with symptoms suggestive of stroke (focal neurological symptoms with sudden onset) referring to Poursina Hospital diagnosed by a neurologist in departments of neurology, emergency and trauma in 2013 were included in the study. 

Patients presenting with subacute or chronic neurological symptoms were excluded. Data collection tool was a checklist containing age, sex, imaging results (brain MRI including T1 weighted, T2 weighted and FLAIR images, and brain CT), early diagnosis and final diagnosis. After collecting and recording, data were analyzed using SPSS Version 21 and the descriptive and inferential statistics (chi-square test). 

To determine the stroke mimics admitted to this hospital, frequency and 95% confidence interval were used.

## Results

In this study, 1985 patients with primary diagnosis of brain stroke, referring to the emergency room of Poursina Hospital were studied. Of all participants, 56.1% (1113) were females and 43.9% (872) were males. Of these, 1690 (85.1%) patients were diagnosed with brain stroke and the rest with stroke mimics (295 patients, 14.9%). In both groups, the ratio of women was more than the men. The proportion of female patients in the stroke mimic group was 1 higher than the other group (56.9 versus 55.9%). Chi-square test showed no significant relationship between sex and early diagnosis of mimic or real stroke. In terms of age groups, the highest percentage belonged to 61-80 years old in both patient groups (real=68.8%, mimics=58.3%). Chi-square test showed a significant correlation between age of patients and early diagnosis of real stroke or stroke mimic (p<0.0001) (table 1).

**Figure 1:**
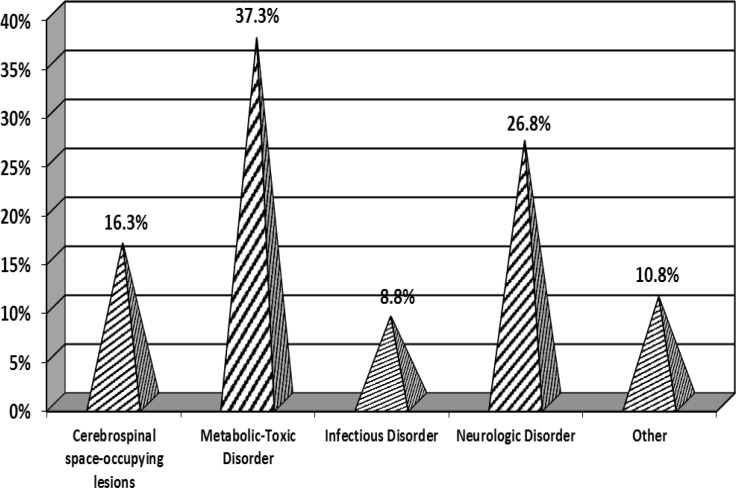
Relative frequency distribution of different general forms of diagnosis for brain stroke mimics in patients with brain stroke symptoms referring to Poursina Hospital, Rasht (2013

The mean age of patients with mimics and brain strokes were 66.5 and 72.4, respectively. There was a significant difference in the mean age of these groups (p<0.0001). In general in the classification of stroke mimics, the highest frequency occurred in patients who (37.2%) suffered from toxic metabolic disorders, whereas the least frequency occurred in patients with infectious diseases (8.8%) (figure 1). 

**Table 1:** Frequency distribution of patients referring to Poursina Hospital with real brain stroke and stroke mimics

**Group**	**Stroke Mimics (n=295)** **Frequency**	**Stroke (n=1690)** **Frequency**	**Pvalue**
**Sex**			
Female Male	168 (56.9) 127 (43.1)	945 (55.9) 745 (44.1)	0.74
**Age (year)**			
<40 41-60 61-80 >80	39 (13.2) 32 (10.8) 172 (58.3) 52 (17.6)	0 (0) 186 (11) 1162 (68.8) 342 (20.2)	0.0001

Moreover, a more detailed examination of the frequency distribution of the different diagnoses for stroke mimics were classified. The highest number belonged to brain tumors (10.5%, 31 patients) contrary to migraine with (1%, 3 patients) (table 2).

**Table 2:** Relative frequency distribution of different diagnoses for brain stroke mimics in patients with clinical signs of brain stroke referring to Poursina Hospital in (2013

**Frequency (%)**	**Diagnosis**
31 (10.5)	Brain Tumors
27 (9.2)	Hypoglycemia
25 (8.5)	Toxic poisoning
24 (8.1)	Hysterical attacks
23 (7.8)	Seizure
22 (7.5)	Sepsis
19 (6.4)	Subdural hematoma
17 (5.8)	Uremia
16 (5.4)	Vestibulopathy
13(4.4)	Multiple sclerosis
12 (4.1)	Brain Metastasis
12 (4)	Encephalopathy
9 (3.1)	Hyperglycemia
9 (3.1)	Syncope
8 (2.7)	Hyponatremia
7 (2.4)	Hyperkalemia
5 (1.7)	Spinal cord Lesions
5 (1.7)	Hypothyroidism
4 (1.4)	Encephalitis
4 (1.4)	Brain Dementia
3 (1)	Migraine
295 (100)	Total

## Discussion

In the present study, a total of 1985 patients were studied, among them 14.9% were inflicted with mimics of brain stroke and the rest (85.1%) were treated after the final diagnosis of stroke. Merino et al.’s study on a 10-year data from the National Institutes of Health Stroke Program found that almost one third of the patients examined by the stroke team experts had stroke mimics (30%) (7). A study conducted in a stroke center revealed that 26.8% of patients referring to the emergency department for stroke had no cerebrovascular problem and even the proportion of hospitalized patients with mimic stroke was higher (8). Of course, there are other studies that are similar to our study with less number of mimics (9) which might be due to equipment such as CT and MRI or setting of study which was a specialized center where patients are evaluated by neurologists. We, in a general classification, found that most of stroke mimics were metabolic and neurological disorders. Furthermore, in a more detailed classification, the highest percentage of mimics was related to brain tumors and the lowest to migraine. The study by Hatzitolios et al. also showed that most patients had neoplastic lesions or primary brain tumor (4). Wolf et al. in their research in 2012 showed that among the 42 patients diagnosed with stroke mimics, 20 had seizure and 7 conversion disorder, 6 dementia, 3 migraine, 2 with brain tumor and 4 with other groups of mimics (10). In a comparison of sex between the two groups, results showed that the number of women was higher than men in both groups of stroke mimics and real stroke. In the study by Hand et al. 50% of patients in stroke group and 45% of patients in stroke mimics were men (10). Other studies also showed that the highest percentage of mimics were observed among women (7, 11). Winkler et al.’s study demonstrated no significant difference between the two groups in terms of gender (12). 

In the current study, the mean age of stroke mimic patients was 66.5±16.39 and the patients with real stroke was 72.4±9.58 years. A statistically significant difference was seen between the groups. There was no stroke in those less than 40 years old and 61 to 80 years group had the highest frequency. In a study by Hand et al., the mean age of brain stroke patients and patients with stroke mimics were 76.3, 76.3 and 77, respectively (13). Gioia et al. also showed that the mean age of patients included in the study was 70.4±15.6 years old (14). While the study by Vroomen et al. showed that the prevalence of brain stroke mimics was very rare in those aged over 50 years (15). Similarly, Winkler et al. stated that no significant difference existed between the different age groups in two groups (12).

In conclusion in the present study, 14.9% of patients had brain stroke mimics. Paying more attention to clinical examinations as the first step can be of great help for the proper diagnosis of brain stroke mimics. Moreover, imaging and laboratory studies have great importance in differentiating stroke from mimics.
